# 1,5-Bis(4-nitro­phen­oxy)penta­ne

**DOI:** 10.1107/S1600536809011672

**Published:** 2009-04-02

**Authors:** Muhammad Saif Ullah Khan, Zareen Akhter, Michael Bolte, M. Saeed Butt, Humaira M. Siddiqi

**Affiliations:** a Department of Chemistry, Quaid-i-Azam University, Islamabad 45320, Pakistan; b Institut für Anorganische Chemie, J. W. Goethe-Universität Frankfurt, Max-von-Laue-Strasse 7, 60438 Frankfurt/Main, Germany

## Abstract

The title compound, C_17_H_18_N_2_O_6_, crystallizes with two mol­ecules in the asymmetric unit. In both molecules, one of the C—C bonds of the penta­methyl­ene chain connecting the two aromatic rings is in a *trans* conformation and another displays a *gauche* conformation. The aromatic rings within each mol­ecule are nearly coplanar [dihedral angles = 3.36 (9) and 4.50 (9)°] and the nitro groups are twisted slightly out of the planes of their attached rings [dihedral angles = 8.16 (3)/6.6 (2) and 4.9 (4)/3.8 (3)°].

## Related literature

For general background and synthetic aspects of thermally stable polymers, see: Critchley *et al.* (1983 ▶); Schab-Balcerzak *et al.* (2002 ▶); Hsiao & Leu (2004 ▶); Hsiao *et al.* (2004 ▶); Mehdipour-Ataei (2005 ▶); Mehdipour-Ataei *et al.* (2006 ▶); Shao *et al.* (2007 ▶); Faghihi (2008 ▶).
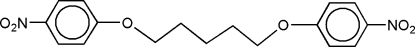


## Experimental

### 

#### Crystal data

C_17_H_18_N_2_O_6_
*M* *_r_* = 346.33
Triclinic, 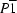

*a* = 10.6032 (8) Å
*b* = 10.7227 (9) Å
*c* = 16.3124 (14) Å
α = 95.603 (7)°
β = 105.718 (6)°
γ = 110.572 (6)°
*V* = 1632.7 (2) Å^3^
*Z* = 4
Mo *K*α radiation
μ = 0.11 mm^−1^
*T* = 173 K
0.27 × 0.24 × 0.19 mm

#### Data collection

Stoe IPDSII two-circle diffractometer
Absorption correction: none
27840 measured reflections
6109 independent reflections
4466 reflections with *I* > 2σ(*I*)
*R*_int_ = 0.056

#### Refinement

*R*[*F* ^2^ > 2σ(*F* ^2^)] = 0.040
*wR*(*F* ^2^) = 0.101
*S* = 0.95
6109 reflections
452 parameters
H-atom parameters constrained
Δρ_max_ = 0.27 e Å^−3^
Δρ_min_ = −0.20 e Å^−3^

### 

Data collection: *X-AREA* (Stoe & Cie, 2001 ▶); cell refinement: *X-AREA*; data reduction: *X-AREA*; program(s) used to solve structure: *SHELXS97* (Sheldrick, 2008 ▶); program(s) used to refine structure: *SHELXL97* (Sheldrick, 2008 ▶); molecular graphics: *PLATON* (Spek, 2009 ▶); software used to prepare material for publication: *SHELXL97*.

## Supplementary Material

Crystal structure: contains datablocks I, global. DOI: 10.1107/S1600536809011672/tk2408sup1.cif

Structure factors: contains datablocks I. DOI: 10.1107/S1600536809011672/tk2408Isup2.hkl

Additional supplementary materials:  crystallographic information; 3D view; checkCIF report

## Figures and Tables

**Fig. 1.**
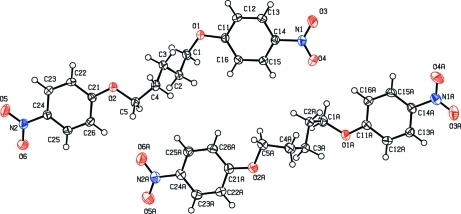
Molecular structure of (I), showing the atom labelling scheme. The displacement ellipsoids are drawn at the 50% probability level.

## supplementary crystallographic information

### Comment

Thermally stable polymers, such as polyamides and polyimides, are an important class of high-performance polymers which are currently receiving considerable interest due to the increasing demands for high-temperature polymers as replacements for metals or ceramics in the automotive, aerospace and microelectronics industries (Critchley *et al.*, 1983). In general, these polymers possess good chemical resistance, low flammability and excellent mechanical properties besides their extraordinary thermal stability (Mehdipour-Ataei *et al.*, 2006). However, they encounter processing difficulties due to their limited solubility in organic solvents and high glass transition (Tg) or melting temperatures (Tm) caused by chain stiffness and intermolecular hydrogen bonding (Hsiao and Leu, 2004). Therefore, much research in the area of thermally-stable polymers in recent years has focused on improving their processability and solubility through the design and synthesis of new monomers (Faghihi, 2008). Aromatic diamines are valuable building blocks for the preparation of thermally-stable polymers which are conventionally used to produce desired alterations in the chemical nature of the macro chain (Mehdipour-Ataei, 2005). Ether linkages are the most popular flexible linkages introduced in the polymer backbone by structural modification of aromatic diamines (Shao *et al.*, 2007). It has been generally recognized that the aryl-ether linkage imparts properties such as better solubility and melt processing characteristics (Hsiao *et al.*, 2004). Moreover, the long flexible aliphatic chains can also be incorporated into the aromatic backbone which effectively disrupt the intermolecular interactions between the aromatic moiety responsible for their high glass transition (Tg) temperatures (Schab-Balcerzak *et al.*, 2002). The title dinitro compound, (I), is a precursor for aromatic diamines and was synthesized as an attempt to design and prepare new monomers for processable high performance polymers, in which the methylene spacers are present between the aromatic rings connected by ether moiety.

Compound (I) crystallizes with two molecules in the asymmetric unit, Fig. 1. One C—C bond of the methylene chain connecting the two aromatic rings is in a *trans* conformation whereas aoother displays a *gauche* conformation so that the molecule has an overall anti-conformation of the aromatic rings. The aromatic rings in a molecule are nearly coplanar [dihedral angles: 3.36 (9)° and 4.50 (9)°] and the nitro groups are are not significantly twisted out of the plane of the ring to which they are attached [dihedral angles: 8.16 (3)°, 6.6 (2)°; 4.9 (4)° and 3.8 (3)°].

### Experimental

A three-necked round bottom flask equipped with Dean-Stark trap, thermometer, magnetic stirrer and nitrogen inlet was charged with a suspension of 1,5-pentane diol (2 ml, 19.1 mmol) and anhydrous potassium carbonate (5.3 g, 38.2 mmol) in a mixture of N, *N*'-dimethyl formamide (DMF) (60 ml) and toluene (20 ml), and refluxed (at 403–408 K) for 2 h for azeotropic removal of water. After cooling to 333–343 K, 1-fluoro-4-nitro benzene (4.05 ml, 38.2 mmol) was added and the mixture was again refluxed for 6 h. Subsequently, some toluene was distilled off and the resulting mixture was poured into 500 ml of chilled water after cooling to room temperature. The crude product was filtered as a yellow solid mass, washed thoroughly with water, dissolved in ethanol and set aside for crystallization. Yield 81%, m.p. 370 K. Calculated C, 58.96, H, 5.24, N, 8.09. C_17_H_18_N_2_O_6_ requires C, 58.70, H, 5.20, N, 7.99. IR (KBr pellet) in cm^-1^: 1581 (aromatic C=C), 1511 and 1341 (NO_2_), 1242 (C=O-C), 2925 (C—H aliphatic), 3078 (C—H aromatic). ^1^H NMR (CDCl_3_) δ: 8.19 (d, 4H, J = 3.1 Hz), 6.98 (d, 4H, J = 3.1 Hz), 4.11 (m, 4H), 1.71 (m, 4H), 1.3 (m, 2H) p.p.m. ^13^C NMR (CDCl_3_) δ: 164.06 (2 C, C4), 141.38 (2 C, C1), 125.96 (4 C, C2,2'), 114.38 (4 C, C3,3'), 68.51 (2 C, C5), 28.73 (2 C, C6), 22.64 (1 C, C7) p.p.m.

### Refinement

All H atoms could be located by difference Fourier synthesis and refined using a riding model with C—H(aromatic) = 0.95 Å or C—H(methylene) = 0.99 Å, and with U(H) = 1.2 *U*_eq_(C).

### Crystal data

**Table:**

C_17_H_18_N_2_O_6_	*Z* = 4
*M**_r_* = 346.33	*F*(000) = 728
Triclinic, *P*1	*D*_x_ = 1.409 Mg m^−^^3^
Hall symbol: -P 1	Mo *K*α radiation, λ = 0.71073 Å
*a* = 10.6032 (8) Å	Cell parameters from 22239 reflections
*b* = 10.7227 (9) Å	θ = 3.6–25.8°
*c* = 16.3124 (14) Å	µ = 0.11 mm^−^^1^
α = 95.603 (7)°	*T* = 173 K
β = 105.718 (6)°	Block, yellow
γ = 110.572 (6)°	0.27 × 0.24 × 0.19 mm
*V* = 1632.7 (2) Å^3^	

### Data collection

**Table:**

Stoe IPDSII two-circle diffractometer	4466 reflections with *I* > 2σ(*I*)
Radiation source: fine-focus sealed tube	*R*_int_ = 0.056
graphite	θ_max_ = 25.7°, θ_min_ = 3.6°
ω scans	*h* = −12→12
27840 measured reflections	*k* = −13→13
6109 independent reflections	*l* = −19→18

### Refinement

**Table:**

Refinement on *F*^2^	Secondary atom site location: difference Fourier map
Least-squares matrix: full	Hydrogen site location: inferred from neighbouring sites
*R*[*F*^2^ > 2σ(*F*^2^)] = 0.040	H-atom parameters constrained
*wR*(*F*^2^) = 0.101	*w* = 1/[σ^2^(*F*_o_^2^) + (0.0621*P*)^2^] where *P* = (*F*_o_^2^ + 2*F*_c_^2^)/3
*S* = 0.95	(Δ/σ)_max_ = 0.001
6109 reflections	Δρ_max_ = 0.27 e Å^−^^3^
452 parameters	Δρ_min_ = −0.20 e Å^−^^3^
0 restraints	Extinction correction: *SHELXL97* (Sheldrick, 2008), Fc^*^=kFc[1+0.001xFc^2^λ^3^/sin(2θ)]^-1/4^
Primary atom site location: structure-invariant direct methods	Extinction coefficient: 0.0093 (10)

### Special details

**Table:**

Geometry. All e.s.d.'s (except the e.s.d. in the dihedral angle between two l.s. planes) are estimated using the full covariance matrix. The cell e.s.d.'s are taken into account individually in the estimation of e.s.d.'s in distances, angles and torsion angles; correlations between e.s.d.'s in cell parameters are only used when they are defined by crystal symmetry. An approximate (isotropic) treatment of cell e.s.d.'s is used for estimating e.s.d.'s involving l.s. planes.
Refinement. Refinement of *F*^2^ against ALL reflections. The weighted *R*-factor *wR* and goodness of fit *S* are based on *F*^2^, conventional *R*-factors *R* are based on *F*, with *F* set to zero for negative *F*^2^. The threshold expression of *F*^2^ > σ(*F*^2^) is used only for calculating *R*-factors(gt) *etc*. and is not relevant to the choice of reflections for refinement. *R*-factors based on *F*^2^ are statistically about twice as large as those based on *F*, and *R*- factors based on ALL data will be even larger.

### Fractional atomic coordinates and isotropic or equivalent isotropic displacement parameters (Å^2^)

**Table:**

	*x*	*y*	*z*	*U*_iso_*/*U*_eq_	
N1	0.65809 (14)	0.79078 (14)	0.53323 (9)	0.0288 (3)	
N2	−0.02055 (14)	−0.37072 (14)	−0.31249 (9)	0.0295 (3)	
O1	0.57451 (11)	0.32831 (11)	0.30135 (7)	0.0286 (3)	
O2	0.31698 (11)	0.12186 (11)	−0.07325 (7)	0.0287 (3)	
O3	0.77971 (13)	0.85858 (13)	0.58276 (9)	0.0494 (4)	
O4	0.55492 (13)	0.81599 (13)	0.53699 (8)	0.0406 (3)	
O5	0.03989 (14)	−0.43169 (14)	−0.34317 (10)	0.0530 (4)	
O6	−0.15085 (12)	−0.41577 (13)	−0.33129 (9)	0.0484 (4)	
C1	0.43378 (17)	0.22437 (16)	0.25233 (11)	0.0286 (4)	
H1A	0.3728	0.2106	0.2899	0.034*	
H1B	0.4426	0.1371	0.2363	0.034*	
C2	0.36236 (16)	0.26221 (16)	0.17000 (10)	0.0261 (3)	
H2A	0.3462	0.3452	0.1865	0.031*	
H2B	0.2682	0.1875	0.1389	0.031*	
C3	0.45014 (16)	0.28805 (16)	0.10867 (10)	0.0255 (3)	
H3A	0.4656	0.2047	0.0919	0.031*	
H3B	0.5447	0.3621	0.1401	0.031*	
C4	0.37983 (18)	0.32734 (16)	0.02630 (11)	0.0282 (4)	
H4A	0.3453	0.3975	0.0429	0.034*	
H4B	0.4524	0.3686	−0.0014	0.034*	
C5	0.25609 (17)	0.20869 (16)	−0.03978 (10)	0.0272 (4)	
H5A	0.1877	0.1581	−0.0118	0.033*	
H5B	0.2054	0.2416	−0.0875	0.033*	
C11	0.58649 (16)	0.44199 (16)	0.35365 (10)	0.0238 (3)	
C12	0.72609 (16)	0.53377 (16)	0.40132 (10)	0.0266 (4)	
H12	0.8044	0.5165	0.3934	0.032*	
C13	0.75079 (16)	0.64867 (16)	0.45955 (10)	0.0262 (4)	
H13	0.8453	0.7100	0.4926	0.031*	
C14	0.63441 (16)	0.67294 (16)	0.46883 (10)	0.0230 (3)	
C15	0.49555 (16)	0.58530 (16)	0.42069 (10)	0.0251 (3)	
H15	0.4179	0.6050	0.4275	0.030*	
C16	0.47045 (16)	0.46922 (17)	0.36281 (10)	0.0256 (3)	
H16	0.3757	0.4086	0.3297	0.031*	
C21	0.22745 (16)	0.00289 (15)	−0.13106 (10)	0.0221 (3)	
C22	0.29377 (16)	−0.07489 (16)	−0.16129 (10)	0.0251 (3)	
H22	0.3947	−0.0437	−0.1405	0.030*	
C23	0.21347 (16)	−0.19676 (16)	−0.22117 (10)	0.0244 (3)	
H23	0.2582	−0.2497	−0.2421	0.029*	
C24	0.06549 (16)	−0.24066 (15)	−0.25036 (10)	0.0232 (3)	
C25	−0.00205 (16)	−0.16517 (16)	−0.22074 (10)	0.0253 (3)	
H25	−0.1031	−0.1972	−0.2415	0.030*	
C26	0.07830 (16)	−0.04292 (16)	−0.16082 (10)	0.0246 (3)	
H26	0.0330	0.0095	−0.1400	0.029*	
N1A	0.54060 (16)	−0.31658 (15)	0.21094 (10)	0.0346 (3)	
N2A	1.20328 (15)	0.86604 (14)	1.04941 (9)	0.0327 (3)	
O1A	0.87307 (12)	0.17493 (12)	0.45211 (8)	0.0336 (3)	
O2A	1.01166 (12)	0.36424 (12)	0.82421 (8)	0.0339 (3)	
O3A	0.60452 (16)	−0.37664 (13)	0.18267 (9)	0.0492 (4)	
O4A	0.40930 (14)	−0.35813 (14)	0.18648 (10)	0.0552 (4)	
O5A	1.33125 (14)	0.92314 (13)	1.09057 (10)	0.0515 (4)	
O6A	1.11687 (14)	0.91610 (13)	1.05638 (9)	0.0468 (3)	
C1A	0.81597 (19)	0.26143 (18)	0.49024 (11)	0.0357 (4)	
H1A1	0.7313	0.2608	0.4454	0.043*	
H1A2	0.8884	0.3562	0.5094	0.043*	
C2A	0.77413 (18)	0.21537 (18)	0.56708 (11)	0.0330 (4)	
H2A1	0.6913	0.1267	0.5460	0.040*	
H2A2	0.7441	0.2822	0.5938	0.040*	
C3A	0.89405 (17)	0.20015 (17)	0.63675 (11)	0.0310 (4)	
H3A1	0.9796	0.2867	0.6549	0.037*	
H3A2	0.9190	0.1279	0.6115	0.037*	
C4A	0.85264 (19)	0.16410 (17)	0.71675 (11)	0.0353 (4)	
H4A1	0.7540	0.0942	0.6973	0.042*	
H4A2	0.9164	0.1242	0.7496	0.042*	
C5A	0.86172 (17)	0.28602 (17)	0.77660 (11)	0.0314 (4)	
H5A1	0.8204	0.3419	0.7423	0.038*	
H5A2	0.8087	0.2560	0.8174	0.038*	
C11A	0.78285 (16)	0.05361 (16)	0.39709 (10)	0.0258 (3)	
C12A	0.85049 (17)	−0.02265 (16)	0.36651 (11)	0.0277 (4)	
H12A	0.9515	0.0095	0.3875	0.033*	
C13A	0.77170 (17)	−0.14412 (16)	0.30615 (11)	0.0275 (4)	
H13A	0.8176	−0.1957	0.2851	0.033*	
C14A	0.62403 (16)	−0.18964 (16)	0.27669 (10)	0.0263 (4)	
C15A	0.55471 (17)	−0.11749 (17)	0.30825 (11)	0.0301 (4)	
H15A	0.4535	−0.1518	0.2885	0.036*	
C16A	0.63379 (17)	0.00471 (17)	0.36867 (11)	0.0297 (4)	
H16A	0.5874	0.0548	0.3906	0.036*	
C21A	1.04962 (17)	0.48516 (16)	0.87813 (10)	0.0269 (4)	
C22A	1.19642 (17)	0.55583 (17)	0.92204 (11)	0.0288 (4)	
H22A	1.2611	0.5176	0.9127	0.035*	
C23A	1.24742 (17)	0.67976 (17)	0.97833 (11)	0.0291 (4)	
H23A	1.3467	0.7274	1.0083	0.035*	
C24A	1.15071 (17)	0.73425 (16)	0.99063 (10)	0.0266 (3)	
C25A	1.00507 (17)	0.66522 (17)	0.94794 (11)	0.0289 (4)	
H25A	0.9409	0.7039	0.9574	0.035*	
C26A	0.95370 (17)	0.54025 (17)	0.89175 (11)	0.0286 (4)	
H26A	0.8542	0.4923	0.8627	0.034*	

### Atomic displacement parameters (Å^2^)

**Table:**

	*U*^11^	*U*^22^	*U*^33^	*U*^12^	*U*^13^	*U*^23^
N1	0.0323 (8)	0.0235 (7)	0.0290 (8)	0.0099 (6)	0.0103 (6)	0.0016 (6)
N2	0.0262 (7)	0.0258 (7)	0.0340 (8)	0.0088 (6)	0.0099 (6)	0.0003 (6)
O1	0.0272 (6)	0.0306 (6)	0.0252 (6)	0.0140 (5)	0.0044 (5)	−0.0038 (5)
O2	0.0264 (6)	0.0260 (6)	0.0296 (6)	0.0111 (5)	0.0053 (5)	−0.0032 (5)
O3	0.0326 (7)	0.0397 (8)	0.0539 (9)	0.0042 (6)	0.0039 (6)	−0.0188 (7)
O4	0.0407 (7)	0.0421 (8)	0.0426 (8)	0.0238 (6)	0.0135 (6)	−0.0027 (6)
O5	0.0361 (7)	0.0392 (8)	0.0727 (10)	0.0107 (6)	0.0185 (7)	−0.0225 (7)
O6	0.0221 (7)	0.0429 (8)	0.0631 (9)	0.0053 (6)	0.0068 (6)	−0.0153 (7)
C1	0.0294 (8)	0.0233 (8)	0.0276 (9)	0.0072 (7)	0.0075 (7)	−0.0012 (7)
C2	0.0243 (8)	0.0261 (8)	0.0236 (8)	0.0085 (7)	0.0057 (6)	−0.0018 (7)
C3	0.0239 (8)	0.0241 (8)	0.0247 (8)	0.0079 (6)	0.0058 (6)	0.0008 (7)
C4	0.0340 (9)	0.0214 (8)	0.0288 (9)	0.0111 (7)	0.0104 (7)	0.0035 (7)
C5	0.0320 (9)	0.0259 (8)	0.0255 (9)	0.0157 (7)	0.0076 (7)	0.0020 (7)
C11	0.0280 (8)	0.0255 (8)	0.0194 (8)	0.0131 (7)	0.0072 (6)	0.0034 (7)
C12	0.0212 (8)	0.0306 (9)	0.0304 (9)	0.0128 (7)	0.0093 (7)	0.0056 (7)
C13	0.0189 (8)	0.0257 (8)	0.0279 (9)	0.0050 (6)	0.0044 (6)	0.0025 (7)
C14	0.0257 (8)	0.0231 (8)	0.0215 (8)	0.0105 (6)	0.0086 (6)	0.0050 (6)
C15	0.0219 (8)	0.0297 (9)	0.0256 (9)	0.0127 (7)	0.0082 (7)	0.0042 (7)
C16	0.0207 (8)	0.0304 (9)	0.0220 (8)	0.0093 (7)	0.0045 (6)	0.0001 (7)
C21	0.0242 (8)	0.0215 (8)	0.0186 (8)	0.0079 (6)	0.0051 (6)	0.0045 (6)
C22	0.0191 (7)	0.0288 (9)	0.0280 (9)	0.0108 (7)	0.0073 (6)	0.0049 (7)
C23	0.0250 (8)	0.0254 (8)	0.0278 (9)	0.0139 (7)	0.0111 (7)	0.0054 (7)
C24	0.0233 (8)	0.0220 (8)	0.0224 (8)	0.0067 (6)	0.0083 (6)	0.0026 (6)
C25	0.0203 (8)	0.0285 (9)	0.0264 (8)	0.0093 (7)	0.0077 (6)	0.0042 (7)
C26	0.0267 (8)	0.0276 (9)	0.0264 (8)	0.0159 (7)	0.0122 (7)	0.0066 (7)
N1A	0.0377 (9)	0.0278 (8)	0.0303 (8)	0.0076 (7)	0.0062 (7)	0.0052 (6)
N2A	0.0353 (8)	0.0273 (8)	0.0326 (8)	0.0078 (7)	0.0129 (7)	0.0060 (6)
O1A	0.0293 (6)	0.0302 (6)	0.0327 (7)	0.0085 (5)	0.0057 (5)	−0.0063 (5)
O2A	0.0305 (6)	0.0305 (6)	0.0343 (7)	0.0116 (5)	0.0042 (5)	−0.0009 (5)
O3A	0.0594 (9)	0.0338 (7)	0.0463 (8)	0.0136 (7)	0.0161 (7)	−0.0056 (6)
O4A	0.0314 (7)	0.0448 (8)	0.0603 (9)	0.0009 (6)	−0.0041 (6)	−0.0088 (7)
O5A	0.0342 (7)	0.0367 (8)	0.0612 (9)	0.0012 (6)	0.0048 (7)	−0.0081 (7)
O6A	0.0459 (8)	0.0386 (8)	0.0546 (9)	0.0169 (6)	0.0185 (7)	−0.0028 (6)
C1A	0.0415 (10)	0.0278 (9)	0.0338 (10)	0.0175 (8)	0.0041 (8)	−0.0012 (8)
C2A	0.0326 (9)	0.0347 (10)	0.0302 (9)	0.0197 (8)	0.0027 (7)	−0.0027 (8)
C3A	0.0301 (9)	0.0284 (9)	0.0323 (9)	0.0157 (7)	0.0035 (7)	−0.0006 (7)
C4A	0.0377 (10)	0.0270 (9)	0.0356 (10)	0.0130 (8)	0.0035 (8)	0.0054 (8)
C5A	0.0299 (9)	0.0299 (9)	0.0288 (9)	0.0087 (7)	0.0056 (7)	0.0044 (7)
C11A	0.0248 (8)	0.0267 (9)	0.0229 (8)	0.0082 (7)	0.0062 (7)	0.0046 (7)
C12A	0.0217 (8)	0.0303 (9)	0.0295 (9)	0.0102 (7)	0.0060 (7)	0.0063 (7)
C13A	0.0286 (8)	0.0262 (9)	0.0299 (9)	0.0132 (7)	0.0099 (7)	0.0054 (7)
C14A	0.0287 (8)	0.0218 (8)	0.0230 (8)	0.0056 (7)	0.0058 (7)	0.0047 (7)
C15A	0.0203 (8)	0.0361 (10)	0.0303 (9)	0.0090 (7)	0.0061 (7)	0.0054 (8)
C16A	0.0260 (8)	0.0358 (9)	0.0285 (9)	0.0145 (7)	0.0092 (7)	0.0024 (7)
C21A	0.0314 (9)	0.0261 (9)	0.0239 (8)	0.0112 (7)	0.0095 (7)	0.0081 (7)
C22A	0.0263 (8)	0.0299 (9)	0.0333 (9)	0.0131 (7)	0.0107 (7)	0.0099 (8)
C23A	0.0240 (8)	0.0301 (9)	0.0307 (9)	0.0072 (7)	0.0086 (7)	0.0095 (7)
C24A	0.0279 (8)	0.0256 (8)	0.0250 (8)	0.0071 (7)	0.0102 (7)	0.0083 (7)
C25A	0.0290 (9)	0.0320 (9)	0.0292 (9)	0.0137 (7)	0.0118 (7)	0.0085 (7)
C26A	0.0221 (8)	0.0324 (9)	0.0282 (9)	0.0089 (7)	0.0059 (7)	0.0065 (7)

### Geometric parameters (Å, °)

**Table:**

N1—O3	1.2273 (18)	N1A—O3A	1.227 (2)
N1—O4	1.2306 (17)	N1A—O4A	1.2347 (19)
N1—C14	1.462 (2)	N1A—C14A	1.463 (2)
N2—O5	1.2217 (18)	N2A—O5A	1.2285 (18)
N2—O6	1.2283 (17)	N2A—O6A	1.2372 (18)
N2—C24	1.461 (2)	N2A—C24A	1.456 (2)
O1—C11	1.3635 (19)	O1A—C11A	1.3583 (19)
O1—C1	1.4556 (19)	O1A—C1A	1.449 (2)
O2—C21	1.3591 (18)	O2A—C21A	1.355 (2)
O2—C5	1.4477 (18)	O2A—C5A	1.4495 (19)
C1—C2	1.521 (2)	C1A—C2A	1.510 (3)
C1—H1A	0.9900	C1A—H1A1	0.9900
C1—H1B	0.9900	C1A—H1A2	0.9900
C2—C3	1.524 (2)	C2A—C3A	1.528 (2)
C2—H2A	0.9900	C2A—H2A1	0.9900
C2—H2B	0.9900	C2A—H2A2	0.9900
C3—C4	1.526 (2)	C3A—C4A	1.528 (2)
C3—H3A	0.9900	C3A—H3A1	0.9900
C3—H3B	0.9900	C3A—H3A2	0.9900
C4—C5	1.515 (2)	C4A—C5A	1.511 (2)
C4—H4A	0.9900	C4A—H4A1	0.9900
C4—H4B	0.9900	C4A—H4A2	0.9900
C5—H5A	0.9900	C5A—H5A1	0.9900
C5—H5B	0.9900	C5A—H5A2	0.9900
C11—C16	1.402 (2)	C11A—C12A	1.399 (2)
C11—C12	1.403 (2)	C11A—C16A	1.400 (2)
C12—C13	1.378 (2)	C12A—C13A	1.380 (2)
C12—H12	0.9500	C12A—H12A	0.9500
C13—C14	1.390 (2)	C13A—C14A	1.389 (2)
C13—H13	0.9500	C13A—H13A	0.9500
C14—C15	1.387 (2)	C14A—C15A	1.390 (2)
C15—C16	1.383 (2)	C15A—C16A	1.385 (2)
C15—H15	0.9500	C15A—H15A	0.9500
C16—H16	0.9500	C16A—H16A	0.9500
C21—C22	1.397 (2)	C21A—C26A	1.397 (2)
C21—C26	1.403 (2)	C21A—C22A	1.404 (2)
C22—C23	1.380 (2)	C22A—C23A	1.374 (2)
C22—H22	0.9500	C22A—H22A	0.9500
C23—C24	1.394 (2)	C23A—C24A	1.394 (2)
C23—H23	0.9500	C23A—H23A	0.9500
C24—C25	1.386 (2)	C24A—C25A	1.390 (2)
C25—C26	1.383 (2)	C25A—C26A	1.382 (2)
C25—H25	0.9500	C25A—H25A	0.9500
C26—H26	0.9500	C26A—H26A	0.9500
			
O3—N1—O4	122.67 (14)	O3A—N1A—O4A	123.12 (15)
O3—N1—C14	118.57 (13)	O3A—N1A—C14A	118.39 (14)
O4—N1—C14	118.73 (13)	O4A—N1A—C14A	118.49 (15)
O5—N2—O6	122.30 (14)	O5A—N2A—O6A	122.39 (15)
O5—N2—C24	118.74 (13)	O5A—N2A—C24A	119.03 (14)
O6—N2—C24	118.94 (13)	O6A—N2A—C24A	118.58 (14)
C11—O1—C1	119.62 (12)	C11A—O1A—C1A	119.74 (12)
C21—O2—C5	118.52 (12)	C21A—O2A—C5A	118.39 (13)
O1—C1—C2	112.48 (13)	O1A—C1A—C2A	112.19 (13)
O1—C1—H1A	109.1	O1A—C1A—H1A1	109.2
C2—C1—H1A	109.1	C2A—C1A—H1A1	109.2
O1—C1—H1B	109.1	O1A—C1A—H1A2	109.2
C2—C1—H1B	109.1	C2A—C1A—H1A2	109.2
H1A—C1—H1B	107.8	H1A1—C1A—H1A2	107.9
C1—C2—C3	112.89 (13)	C1A—C2A—C3A	113.39 (14)
C1—C2—H2A	109.0	C1A—C2A—H2A1	108.9
C3—C2—H2A	109.0	C3A—C2A—H2A1	108.9
C1—C2—H2B	109.0	C1A—C2A—H2A2	108.9
C3—C2—H2B	109.0	C3A—C2A—H2A2	108.9
H2A—C2—H2B	107.8	H2A1—C2A—H2A2	107.7
C2—C3—C4	113.21 (13)	C2A—C3A—C4A	112.27 (14)
C2—C3—H3A	108.9	C2A—C3A—H3A1	109.1
C4—C3—H3A	108.9	C4A—C3A—H3A1	109.1
C2—C3—H3B	108.9	C2A—C3A—H3A2	109.1
C4—C3—H3B	108.9	C4A—C3A—H3A2	109.1
H3A—C3—H3B	107.7	H3A1—C3A—H3A2	107.9
C5—C4—C3	113.82 (13)	C5A—C4A—C3A	112.77 (14)
C5—C4—H4A	108.8	C5A—C4A—H4A1	109.0
C3—C4—H4A	108.8	C3A—C4A—H4A1	109.0
C5—C4—H4B	108.8	C5A—C4A—H4A2	109.0
C3—C4—H4B	108.8	C3A—C4A—H4A2	109.0
H4A—C4—H4B	107.7	H4A1—C4A—H4A2	107.8
O2—C5—C4	106.26 (12)	O2A—C5A—C4A	106.70 (13)
O2—C5—H5A	110.5	O2A—C5A—H5A1	110.4
C4—C5—H5A	110.5	C4A—C5A—H5A1	110.4
O2—C5—H5B	110.5	O2A—C5A—H5A2	110.4
C4—C5—H5B	110.5	C4A—C5A—H5A2	110.4
H5A—C5—H5B	108.7	H5A1—C5A—H5A2	108.6
O1—C11—C16	124.34 (14)	O1A—C11A—C12A	114.50 (13)
O1—C11—C12	115.71 (13)	O1A—C11A—C16A	125.41 (15)
C16—C11—C12	119.94 (14)	C12A—C11A—C16A	120.06 (15)
C13—C12—C11	120.69 (14)	C13A—C12A—C11A	120.56 (15)
C13—C12—H12	119.7	C13A—C12A—H12A	119.7
C11—C12—H12	119.7	C11A—C12A—H12A	119.7
C12—C13—C14	118.57 (15)	C12A—C13A—C14A	118.78 (15)
C12—C13—H13	120.7	C12A—C13A—H13A	120.6
C14—C13—H13	120.7	C14A—C13A—H13A	120.6
C15—C14—C13	121.66 (15)	C13A—C14A—C15A	121.51 (15)
C15—C14—N1	118.87 (13)	C13A—C14A—N1A	118.80 (15)
C13—C14—N1	119.42 (14)	C15A—C14A—N1A	119.69 (14)
C16—C15—C14	119.87 (14)	C16A—C15A—C14A	119.71 (14)
C16—C15—H15	120.1	C16A—C15A—H15A	120.1
C14—C15—H15	120.1	C14A—C15A—H15A	120.1
C15—C16—C11	119.23 (15)	C15A—C16A—C11A	119.33 (15)
C15—C16—H16	120.4	C15A—C16A—H16A	120.3
C11—C16—H16	120.4	C11A—C16A—H16A	120.3
O2—C21—C22	115.35 (13)	O2A—C21A—C26A	124.47 (15)
O2—C21—C26	124.43 (14)	O2A—C21A—C22A	115.47 (14)
C22—C21—C26	120.22 (14)	C26A—C21A—C22A	120.06 (16)
C23—C22—C21	120.40 (14)	C23A—C22A—C21A	120.62 (15)
C23—C22—H22	119.8	C23A—C22A—H22A	119.7
C21—C22—H22	119.8	C21A—C22A—H22A	119.7
C22—C23—C24	118.72 (14)	C22A—C23A—C24A	118.73 (15)
C22—C23—H23	120.6	C22A—C23A—H23A	120.6
C24—C23—H23	120.6	C24A—C23A—H23A	120.6
C25—C24—C23	121.64 (14)	C25A—C24A—C23A	121.31 (16)
C25—C24—N2	119.22 (13)	C25A—C24A—N2A	119.34 (15)
C23—C24—N2	119.13 (14)	C23A—C24A—N2A	119.36 (14)
C26—C25—C24	119.71 (14)	C26A—C25A—C24A	119.96 (15)
C26—C25—H25	120.1	C26A—C25A—H25A	120.0
C24—C25—H25	120.1	C24A—C25A—H25A	120.0
C25—C26—C21	119.31 (14)	C25A—C26A—C21A	119.31 (15)
C25—C26—H26	120.3	C25A—C26A—H26A	120.3
C21—C26—H26	120.3	C21A—C26A—H26A	120.3
			
C11—O1—C1—C2	79.68 (17)	C11A—O1A—C1A—C2A	79.87 (18)
O1—C1—C2—C3	57.73 (17)	O1A—C1A—C2A—C3A	53.61 (19)
C1—C2—C3—C4	−179.48 (13)	C1A—C2A—C3A—C4A	175.62 (14)
C2—C3—C4—C5	−75.13 (17)	C2A—C3A—C4A—C5A	−78.46 (18)
C21—O2—C5—C4	176.62 (12)	C21A—O2A—C5A—C4A	175.20 (13)
C3—C4—C5—O2	−68.71 (17)	C3A—C4A—C5A—O2A	−76.49 (16)
C1—O1—C11—C16	−1.6 (2)	C1A—O1A—C11A—C12A	−178.18 (14)
C1—O1—C11—C12	177.31 (13)	C1A—O1A—C11A—C16A	3.7 (2)
O1—C11—C12—C13	−176.71 (14)	O1A—C11A—C12A—C13A	−176.12 (14)
C16—C11—C12—C13	2.2 (2)	C16A—C11A—C12A—C13A	2.1 (2)
C11—C12—C13—C14	−1.2 (2)	C11A—C12A—C13A—C14A	−0.4 (2)
C12—C13—C14—C15	−0.5 (2)	C12A—C13A—C14A—C15A	−1.6 (2)
C12—C13—C14—N1	177.13 (14)	C12A—C13A—C14A—N1A	178.37 (14)
O3—N1—C14—C15	171.40 (15)	O3A—N1A—C14A—C13A	−1.7 (2)
O4—N1—C14—C15	−6.8 (2)	O4A—N1A—C14A—C13A	178.37 (15)
O3—N1—C14—C13	−6.3 (2)	O3A—N1A—C14A—C15A	178.32 (15)
O4—N1—C14—C13	175.47 (14)	O4A—N1A—C14A—C15A	−1.6 (2)
C13—C14—C15—C16	1.1 (2)	C13A—C14A—C15A—C16A	1.8 (2)
N1—C14—C15—C16	−176.51 (14)	N1A—C14A—C15A—C16A	−178.17 (14)
C14—C15—C16—C11	−0.1 (2)	C14A—C15A—C16A—C11A	0.0 (2)
O1—C11—C16—C15	177.26 (14)	O1A—C11A—C16A—C15A	176.14 (15)
C12—C11—C16—C15	−1.6 (2)	C12A—C11A—C16A—C15A	−1.9 (2)
C5—O2—C21—C22	179.31 (13)	C5A—O2A—C21A—C26A	0.4 (2)
C5—O2—C21—C26	−0.3 (2)	C5A—O2A—C21A—C22A	−179.83 (13)
O2—C21—C22—C23	−179.09 (14)	O2A—C21A—C22A—C23A	179.88 (14)
C26—C21—C22—C23	0.5 (2)	C26A—C21A—C22A—C23A	−0.3 (2)
C21—C22—C23—C24	−0.3 (2)	C21A—C22A—C23A—C24A	−0.3 (2)
C22—C23—C24—C25	0.0 (2)	C22A—C23A—C24A—C25A	0.6 (2)
C22—C23—C24—N2	−178.87 (14)	C22A—C23A—C24A—N2A	−179.41 (14)
O5—N2—C24—C25	174.65 (16)	O5A—N2A—C24A—C25A	176.09 (15)
O6—N2—C24—C25	−6.2 (2)	O6A—N2A—C24A—C25A	−3.5 (2)
O5—N2—C24—C23	−6.5 (2)	O5A—N2A—C24A—C23A	−3.9 (2)
O6—N2—C24—C23	172.66 (15)	O6A—N2A—C24A—C23A	176.52 (15)
C23—C24—C25—C26	0.1 (2)	C23A—C24A—C25A—C26A	−0.2 (2)
N2—C24—C25—C26	178.96 (14)	N2A—C24A—C25A—C26A	179.80 (14)
C24—C25—C26—C21	0.1 (2)	C24A—C25A—C26A—C21A	−0.4 (2)
O2—C21—C26—C25	179.14 (14)	O2A—C21A—C26A—C25A	−179.51 (15)
C22—C21—C26—C25	−0.4 (2)	C22A—C21A—C26A—C25A	0.7 (2)
